# Differentially expression analyses in fruit of cultivated and wild species of grape and peach

**DOI:** 10.1038/s41598-023-29025-w

**Published:** 2023-02-03

**Authors:** Weijian Huang, Meng Li, Huangwei Zhang, Juyou Wu, Jim M. Dunwell, Shaoling Zhang

**Affiliations:** 1 College of Horticulture, Nanjing Agricultural University, Nanjing, 210095 China; 2 College of Agriculture and Bioengineering, Taizhou Vocational College of Science & Technology, Taizhou, 318020 China; 3 School of Agriculture, Policy and Development, University of Reading, Earley Gate, Reading, RG6 6AH UK

**Keywords:** Plant domestication, Transcriptomics

## Abstract

Through agronomic traits and sequencing data, the cultivated and wild varieties of grapes and peaches were analyzed and compared in terms of fruit size, fruit flavor, fruit resistance, and fruit color. Cultivated grapes and peaches have advantages in fruit size, soluble sugar content, sugar and acid ratio, etc. Wild grapes and peaches have utility value in resistance. The results showed that there were 878 and 301 differentially expressed genes in cultivated and wild grapes and peaches in the three growth stages, respectively based on the next-generation sequencing study. Ten and twelve genes related to the differences between cultivated and wild grapes and peaches were found respectively. Among them, three genes, namely chalcone synthase (*CHS*), glutathione S-transferase (*GST*) and malate dehydrogenase (*MDH1*) were present in both cultivated and wild grapes and peaches.

## Introduction

Grapes (*Vitis vinifera* L.) are woody vines of the *Vitaceae* family (*Vitis* L.)^1^. There are currently approximately 70 different grape types grown throughout the world, with the majority found in temperate or subtropical regions of East Asia and North America^2^. In 2021, the world’s grape production exceeded 82 million tons (2021, FAOSTAT). The peach tree (*Prunus persica* L.) is a perennial tree in the rose family^3^. In 2020, China’s peach cultivation area and output both ranked first in the world, with an output exceeding 77,600 tons. Through the improvement of fruit tree varieties and the continuous optimization of cultivation methods, from 2000 to 2021, the production of grapes and peaches in China increased exceeding fourfold (2021, FAOSTAT).

Artificial selection and natural selection have resulted in cultivated and wild grapes and peaches having great differences in fruit traits such as quality, peel color, and flavor. The present study was designed to reveal the associated differences between cultivated and wild grape and peach fruit traits, and to conduct transcriptome analysis to identify differences in gene expression^4^. Such sequencing technology can accurately quantify the expression level of specific genes, and their alleles; this is helpful for in-depth analysis of biological problems^5^.

Much research has been conducted on transcriptome sequencing of grapes and peaches^6–11^. For example, QTL analysis revealed candidate genes (*Ppa004358m*、*Ppa010376m*、*Ppa018828m* and *Ppb018041m*) related to fruit swelling, and discussed the significance of fruit swelling speed and during fruit growth and development and the potential gene regulatory network related to peach fruit. Ye^12^ selected the peel of peach variety ‘Jinxiu’ at three time points (105, 120 and 135 days after flowering) as samples, performed transcriptome sequencing and annotated 25,694 unigenes, 31 genes were found significantly related to anthocyanin content, including 8 enzyme genes (*CHS*、*CHI*、*F3H*、*DFR*、*UFGT*、*4CL*、*FLS* and *F3'H*). This study providesd an important reference for further understanding of peach skin color formation mechanism. Most previous transcriptome studies have focused on the differences under different treatment conditions and there are few studies conducted on the differences between cultivated and wild fruit trees. The regulatory mechanism of domestication research is required because many great genes in wild germplasm resources can be exploited for the domestication and cultivation of cultivated kinds.

In this study, cultivated grapes ‘Pinot Noir’ (*Vitis vinifera* L.) and wild grapes ‘Changbai No. 9’ (*Vitis amurensis* Rur.), cultivated peaches ‘Okubo’ (*Prunus persica* L.) and wild peach ‘Lianyungang Maotao’ (*Amygdalus persica* L.) were selected as the experimental material to study the difference in the fruit traits:- single fruit weight, horizontal diameter, vertical diameter, soluble sugar content and organic acid content. Gene expression of grapes and peaches from typical cultivated and wild fruit trees, and their influence on fruit quality during domestication were investigated. The differences in fruit traits such as size, resistance and peel color were discussed. These findings are of great significance for the utilization of excellent characters in wild fruit tree resources and the optimized breeding of cultivated fruit trees.

## Results

### Transcript sequencing data set

This study included 16 libraries of samples from grapes and peaches at three growth stages (Table 1). The comparative results of grape sample sequencing data are shown in Table 2. A total of 186,132,112 reads were generated, of which C_G_t2 had the largest number of reads with 29,858,769 reads. The least is C_G_t1, with 15,933,645 reads. After comparing the reads to the grape reference genome, the highest similarity frequency is 77.60% (C_G_t1), the average frequency of four samples of cultivated grapes reached 73.15%, and the average similarity frequency of four samples of wild grapes was 59.5%. After assembly, a total of 27,264 genes and 66,713 transcripts were obtained. The length of contig N50 reached 2715 kb, and the average length of contigs reached 1881 kb. The annotation results showed that 44,007 transcripts were completely matched to the reference genome.

**Table 1:** Sample abbreviation specifications.

Species	Sample abbreviation	Sample description
Grape	C_G_t1	Fruit samples of cultivated grape ‘Pinot Noir’ in young fruit stage
	C_G_t2	Fruit samples of cultivated grape ‘Pinot Noir’ in swelling stage
	C_G_t3	Fruit samples of cultivated grape ‘Pinot Noir’ in maturity
	C_G_leaf	Leaf samples of cultivated grape ‘Pinot Noir’ in maturity
	W_G_t1	Fruit samples of wild grape ‘Changbai No. 9’ in young fruit stage
	W_G_t2	Fruit samples of wild grape ‘Changbai No. 9’ in swelling stage
	W_G_t3	Fruit samples of wild grape ‘Changbai No. 9’ in maturity
	W_G_leaf	Leaf samples of wild grape ‘Changbai No. 9’ in maturity
Peach	C_P_t1	Fruit samples of cultivated peach ‘Okubo’ in young fruit stage
	C_P_t2	Fruit samples of cultivated peach ‘Okubo’ in swelling stage
	C_P_t3	Fruit samples of cultivated peach ‘Okubo’ in maturity
	C_P_leaf	Leaf samples of cultivated peach ‘Okubo’ in maturity
	W_P_t1	Fruit samples of wild peach ‘Lianyungang Maotao’ in young fruit stage
	W_P_t2	Fruit samples of wild peach ‘Lianyungang Maotao’ in swelling stage
	W_P_t3	Fruit samples of wild peach ‘Lianyungang Maotao’ in maturity
	W_P_leaf	Leaf samples of wild peach ‘Lianyungang Maotao’ in maturity

**Table 2:** Comparison results of transcript sequencing data.

Species	Sample abbreviation	Total pairs	Mapped reads	Concordant pairs
Grape	C_G_t1	15 933 645	82.80%	77.60%
	C_G_t2	29 858 769	79.30%	73.80%
	C_G_t3	22 781 342	76.60%	70.60%
	C_G_leaf	25 566 026	75.60%	70.60%
	W_G_t1	17 098 859	73.00%	63.40%
	W_G_t2	26 403 616	68.90%	59.50%
	W_G_t3	23 370 214	69.90%	60.60%
	W_G_leaf	25 119 641	62.40%	54.70%
Peach	C_P_t1	24 512 137	85.20%	81.00%
	C_P_t2	17 326 896	88.20%	84.20%
	C_P_t3	30 382 911	88.10%	84.40%
	C_P_leaf	16 055 962	87.60%	83.60%
	W_P_t1	15 770 694	86.50%	82.20%
	W_P_t2	17 409 126	88.20%	84.20%
	W_P_t3	29 320 743	87.10%	84.30%
	W_P_leaf	26 376 610	85.80%	81.50%

The comparative results of the sequencing data of the peach samples are shown in Table 2. A total of 177,155,079 reads were generated, of which C_P_t1 had the largest number of reads with 30,382,911 reads, and the smallest was W_P_t1 with 15,770,694 reads. After comparing reads to the peach reference genome, it was found that the highest similarity frequency was 84.40% (C_P_t1), and the lowest was 81.00% (C_P_t3). The average similarity frequency of the four samples of ‘Okubo’ reached 83.3%, ‘the average similarity frequency of the four samples of ‘Lianyungang Maotao’ is 83.1%. A total of 27,999 genes were obtained after assembly. With 56,876 transcripts, the length of contig N50 reached 2475 kb, and the average length of contigs reached 1635 kb. The annotation results showed that 29,542 transcripts were perfectly matched to the reference genome.

### Differences in transverse diameter, longitudinal diameter and single fruit weight

**Figure 1:**
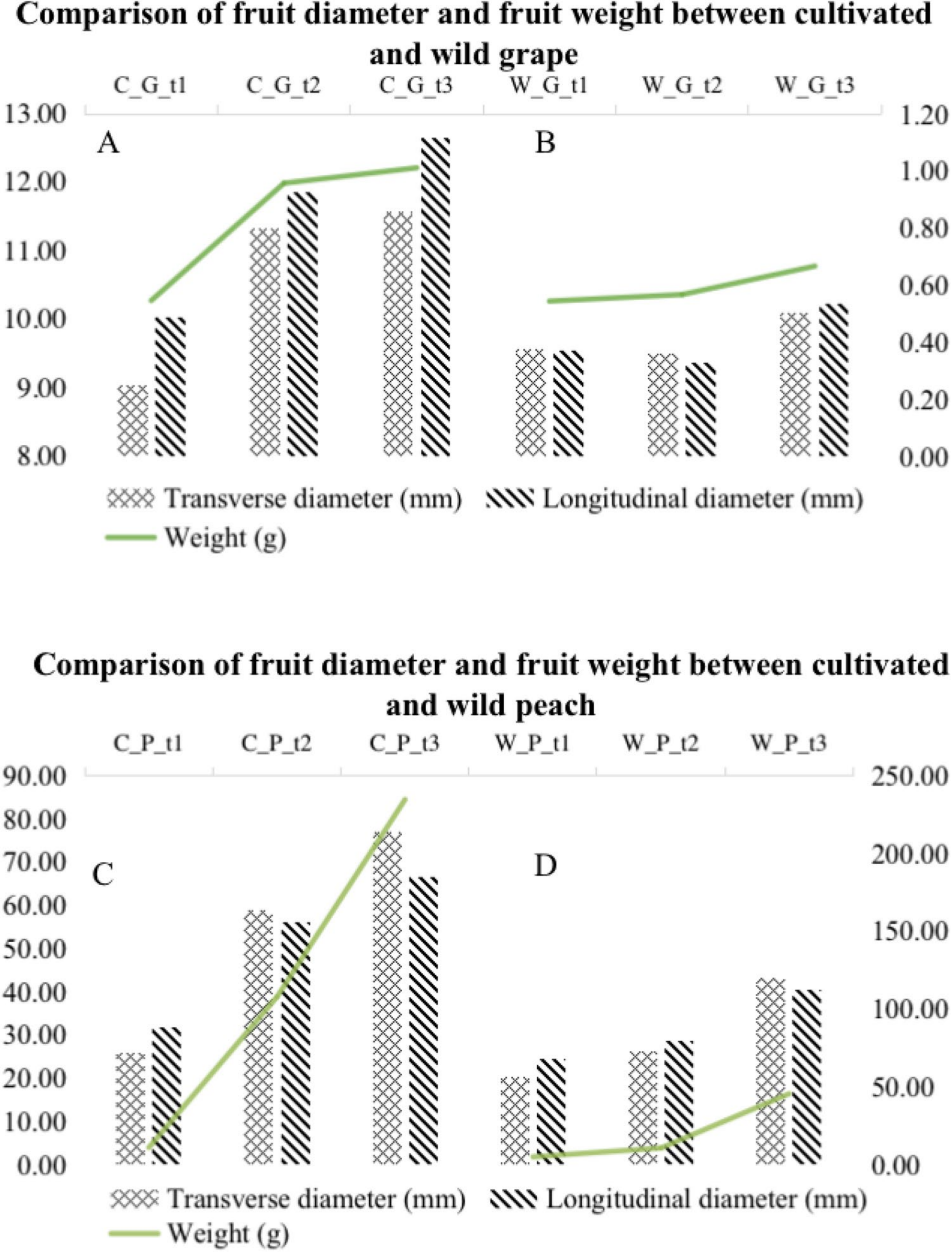
Comparison of fruit diameter and fruit weight between cultivated and wild grape and peach. Changes in the horizontal and vertical diameters and individual fruit weight of cultivated and wild grapes and peaches in the three periods. The top abscissa represents cultivated and wild samples in three periods. The left axis is the horizontal and vertical diameter (mm), and the right axis is the weight of a single fruit (g). The grid column represents the changing trend of the fruit’s horizontal diameter, and the diagonal column represents the fruit’s vertical diameter. The broken line represents the change trend of fruit weight. (**A**) refers to cultivated grape ‘Pinot Noir’, (**B**) refers to wild grape ‘Changbai No. 9’, (**C**) refers to cultivated peach ‘Okubo’, **D** refers to wild peach ‘Lianyungang Maotao’.

In the grape fruits (Fig. 1, Table 3), the horizontal and vertical diameters of cultivated grape ‘Pinot Noir’ have relatively large changes during the three development periods. In the horizontal diameter, the expansion stage of ‘Pinot Noir’ increased by 2.28 mm, which was 1.25 times, compared with the young fruit stage; and the mature stage increased by 0.24 mm, which was 1.02 times, compared with the expansion stage. Also, it increased by 2.5 mm from the young fruit stage to the mature stage, which was 1.28 times. In the longitudinal diameter, the expansion stage of 'Pinot Noir' increased by 1.82 mm, which was 1.18 times compared with the young fruit stage; and the mature stage increased by 0.8 mm, which was 1.07 times, compared with the expansion stage, and it increased by 2.6 mm, which was 1.26 times, from the young fruit stage to the mature stage.

**Table 3:** Sugar and acid content of cultivated and wild grapes and peach fruits in three periods.

Sample		Soluble sugar ($\mathrm{mg}\cdot \mathrm{g}$^−1^)				Organic acid ($\mathrm{mg}\cdot \mathrm{g}$^−1^)				
		Sorbitol	Fructose	Glucose	Sucrose	Quinic	Citric	Malic	Oxalic	Shikimic
Grape	C_G_t1	–	1.98	1.80	–	10.64	–	10.02	0.04	0.03
	C_G_t2	–	56.00	51.02	2.50	1.84	–	1.27	0.05	–
	C_G_t3	–	50.65	45.79	3.11	3.67	0.11	1.34	0.08	–
	W_G_t1	–	1.04	0.11	0.05	11.51	–	4.87	0.01	–
	W_G_t2	–	12.06	9.61	1.01	4.81	0.16	5.54	0.01	0.01
	W_G_t3	–	21.75	18.99	2.80	0.60	0.26	3.54	0.01	0.01
Peach	C_P_t1	5.64	16.38	15.90	4.83	3.01	0.01	3.58	0.22	–
	C_P_t2	6.27	7.38	7.01	20.30	0.77	0.35	1.24	0.10	0.02
	C_P_t3	0.86	9.63	9.56	28.15	1.23	–	2.95	0.31	0.04
	W_P_t1	5.21	3.54	24.03	6.98	6.49	–	8.70	0.73	0.26
	W_P_t2	3.00	2.45	4.67	4.87	3.19	2.28	4.38	0.57	0.13
	W_P_t3	0.92	0.95	3.78	9.45	0.30	–	0.56	0.13	0.01

The horizontal and vertical diameters of the wild grape ‘Changbai No. 9’ had relatively small changes in the three periods. For the horizontal diameter, the expansion stage and young fruit stage of ‘Changbai No. 9’ were basically the same, and the mature stage is 0.61 mm longer than the expansion stage, which was 1.02 times. From the young fruit stage to the mature stage, the increase was 0.54 mm, which was 1.06 times. For the longitudinal diameter, the swelling stage and the young fruit stage of ‘Changbai No. 9’ are basically the same. The mature stage is 0.85 mm, which was 1.09 times longer than the swelling stage, and 0.68 mm, which was 1.07 times from the young fruit stage to the mature stage.

**Figure 2:**
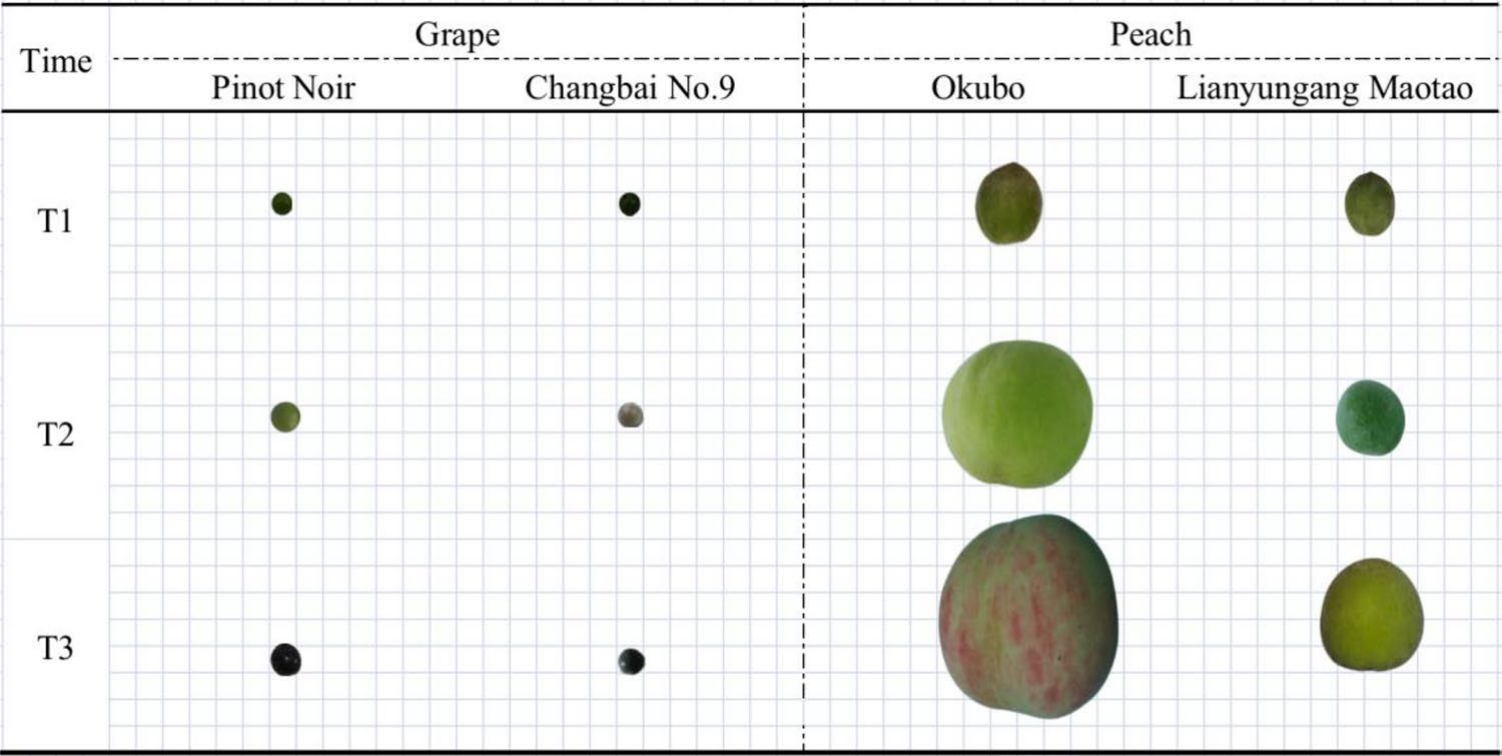
Wild and cultivated fruits in different periods. Comparison of fruit size changes between cultivated and wild grapes and peaches in three periods T1 represents young fruit period, T2 represents swelling period, and T3 represents maturity period.

The cultivated and wild grapes maintain the same trend in fruit weight per fruit. During the three periods, the weight gradually increased with the growth and development of the fruit. The change of fruit weight of the cultivated grape ‘Pinot Noir’ in the three periods is more significant than that of the wild grape ‘Changbai No. 9’ (Fig. 2). The single fruit weight of ‘Pinot Noir’ increased by 0.41 g at swelling period, which was 1.75 times compared with the young fruit period; and the mature period increased by 0.05 g, which was 1.06 and 1.85 times when compared with the swelling period and the young fruit stage respectively. The single fruit weight of the wild grape ‘Changbai No. 9’ had a small change compared with the cultivated grape ‘Pinot Noir’. The single fruit weight of ‘Changbai No. 9’ was basically the same in the swelling stage and the young fruit stage, and the ripening stage was increased by 0.1 g compared with the swelling stage, which was 1.18 times. Also, the mature stage was 0.12 g more than the young fruit stage, which was 1.22 times. The growth rate of the cultivated grape ‘Pinot Noir’ increased significantly compared with the wild grape ‘Changbai No. 9’, especially from the young fruit stage to the expansion stage, while the growth rate of ‘Changbai No. 9’ was more obvious from the expansion stage to the mature stage.

The horizontal and vertical diameters of cultivated peach 'Okubo' varied greatly in the three periods (Fig. 1, Table 3). For the horizontal diameter, the expansion period of wild peach ‘Okubo’ increased by 33.15 mm compared with the young fruit stage, which was 2.28 times. Compared with the swelling stage, the mature period increased by 18.18 mm, which was 1.31 times. Also, the total increase from the young fruit stage to the mature stage was 51.3 mm, which was 2.98 times of the young fruit stage. For the longitudinal diameter, the swelling stage of cultivated peach ‘Okubo’ increased by 24.46 mm, which was 1.77 times, compared with the young fruit stage, while the mature stage increased by 10.15 mm, which was 1.18 times, compared with the swollen stage. Also, the total increase from the young fruit stage to the mature stage increased by 34.61 mm, which was 2.09 times.

In terms of the single fruit weight of peach fruit, the weight of cultivated peach ‘Okubo’ changed significantly in the three stages. The expansion stage increased by 96.85 g, which was 9.79 times, compared with the young fruit stage, and the mature stage increased by 126.97 g, which was 2.18 times, compared with the expansion stage. Compared with the young fruit stage, the mature stage increased by 223.82 g, which was 21.32 times.

The horizontal and vertical diameter changes of the wild peach ‘Lianyungang Maotao’ in the three stages were smaller than that of the ‘Okubo’. For the horizontal diameter, the expansion period of the wild peach ‘Lianyungang Maotao’ increased by 5.8 mm compared with the young fruit stage, which was 2.28 times at the mature stage. Compared with the swelling period, it increased by 17.21 mm, which was 1.31 times. From the young fruit stage to the mature stage, total increase was 23.01 mm, which was 2.99 times. For the longitudinal diameter, the swelling stage of the wild peach ‘Lianyungang Maotao’ increased by 4.24 mm, which was 1.77 times compared with the young fruit stage, and the mature stage increased by 11.62 mm, which was 1.18 times compared with the swollen stage, and the total increase from the young fruit stage to the mature stage increased by 15.85 mm, which was 2.09 times.

The individual fruit weight of the wild peach ‘Lianyungang Peach’ had relatively little change. The mature stage increased by 34.91 g, which was 4.27 times compared with the expansion stage, and the total increased by 40.6 g, which was 9.12 times compared with the young fruit stage.

Combining the above results, it can be seen that the cultivated peach ‘Okubo’ had a higher growth rate than the wild peach ‘Lianyungang Maotao’, and it is dominant in the transverse diameter, longitudinal diameter and single fruit weight, and the skin color of fruit is more red.

### Difference in soluble sugar and organic acid content of fruit

Soluble sugars in grape and peach fruits include sorbitol, fructose, glucose, and sucrose, and organic acids include quinic acid, citric acid, malic acid, oxalic acid, and shikimic acid. As shown in Table 3, the total sugar content of cultivated grape ‘Pinot Noir’ increased from 3.78 mg·g^−1^ to 99.55 mg·g^−1^, with an increase of 95.77 mg·g^−1^ from the first stage to the third stage, while the sugar content of ‘Changbai No.9’ increased from 1.2 mg·g^−1^ to 43.54 mg·g^−1^, with an increase of 42.34 mg·g^−1^. In terms of sugar content, the increase of ‘Pinot Noir’ was 53.43 mg·g^−1^ more than that of ‘Changbai No.9’, which was 2.26 times.

The average sugar-acid ratio of ‘Pinot Noir’ was 4.27 times that of ‘Changbai No.9’, which was 18.13. The average sugar-acid ratio of ‘Changbai No.9’ was 4.25. At maturity stage, the sugar-to-acid ratio of 'Pinot Noir' was 1.86 times that of ‘Changbai No. 9’, reached 19.56, and that of ‘Changbai No. 9’ was 10.49. It can be seen that the sugar content of the cultivated grape ‘Pinot Noir’ in the three periods was higher than that of the wild grape ‘Changbai No.9’. Also, the sugar-acid ratio was higher, which means an advantage in fruit sweetness. For the cultivated grape ‘Pinot Noir’, the content of fructose and glucose changed significantly. From the young fruit stage to the mature stage, the fructose content increased by 48.67 mg·g^−1^ and the glucose content increased by 43.99 mg·g^−1^. From the expansion stage to the mature stage, the sucrose increased to 3.11 mg·g^−1^. Compared with the cultivated grape ‘Pinot Noir’, the wild grape ‘Changbai No.9’ had a small increase in soluble sugar content. From the young fruit stage to the swelling stage, the fructose content increased by 11.02 mg·g^−1^, the glucose increased by 9.5 mg·g^−1^, and the sucrose only increased by 0.96 mg·g^−1^. From the expansion stage to the mature stage, the fructose content increased by 9.69 mg·g^−1^, glucose increased by 9.38 mg·g^−1^, and sucrose increased by 2.8 mg·g^−1^. The content of quinic acid in the cultivated grape ‘Pinot Noir’ decreased from 6.97 mg·g^−1^ to 3.67 mg·g^−1^ from the young fruit stage to the mature stage, and the wild cultivar ‘Changbai No.9’ decreased by 10.91 mg·g^−1^ to 0.6 mg·g^−1^in the same period. The content of malic acid in ‘Pinot Noir’ decreased from 8.68 mg·g^−1^ from the young fruit stage to the mature stage to 1.34 mg·g^−1^. Also, in the same period, the content of ‘Changbai No.9’ decreased from 1.33 mg·g^−1^ to 3.54 mg·g^−1^. The content of oxalic acid in ‘Pinot Noir’ ranged from 0.04 mg·g^−1^ in the young fruit stage to 0.08 mg·g^−1^ in the mature stage, while the content of ‘Changbai No.9’ was maintained at 0.01 mg·g^−1^. In terms of citric acid content, ‘Pinot Noir’ maintained a low level, with a content of 0.11 mg·g^−1^ in the mature stage, and a citric acid content of 0.26 mg·g^−1^ in ‘Changbai No.9’.

The soluble sugar content of cultivated peach 'Okubo' was higher than that of wild peach ‘Lianyungang Maotao’ at every stage, and the organic acid content was lower. The total soluble sugar content of cultivated peach 'Okubo' increased from 42.75 mg·g^−1^ to 48.2 mg·g^−1^, while the total soluble sugar content of wild peach ‘Lianyungang Maotao’ decreased from 39.76 mg·g^−1^ to 15.1 mg·g^−1^. In terms of total soluble sugar content, cultivated peach ‘Okubo’ increased 30.11 mg·g^−1^ more than wild peach ‘Lianyungang Maotao’. The total organic acid content of the cultivated peach ‘Okubo’ increased from 6.82 mg·g^−1^ to 4.53 mg·g^−1^, which increased 2.29 mg·g^−1^, while the total organic acid content of the wild peach ‘Lianyungang Maotao’ decreased from 16.18 mg·g^−1^ to 1 mg·g^−1^. In terms of total organic acid content, the reduction of wild peach ‘Lianyungang Maotao’ is 12.89 mg·g^−1^ more than that of cultivated peach ‘Okubo’. The sucrose content of cultivated peach ‘Okubo’ and wild peach ‘Lianyungang Maotao’ maintained an increasing trend during the three periods. The sucrose content of cultivated peach ‘Okubo’ ranged from 4.83 mg·g^−1^ to 28.18 mg·g^−1^, accounting for 58% of the total soluble sugar content. The sucrose content of wild peach ‘Lianyungang Maotao’ ranges from 6.98 mg·g^−1^ to 9.45 mg·g^−1^, accounting for 62% of the total soluble sugar content, and the content of all other soluble sugars showed a downward trend. The content of quinic acid and malic acid in the cultivated peach ‘Okubo’ and the wild peach ‘Lianyungang Maotao’ decreased significantly in the three periods.

### Analysis of gene differential expression in cultivated and wild grapes and peach

**Figure 3:**
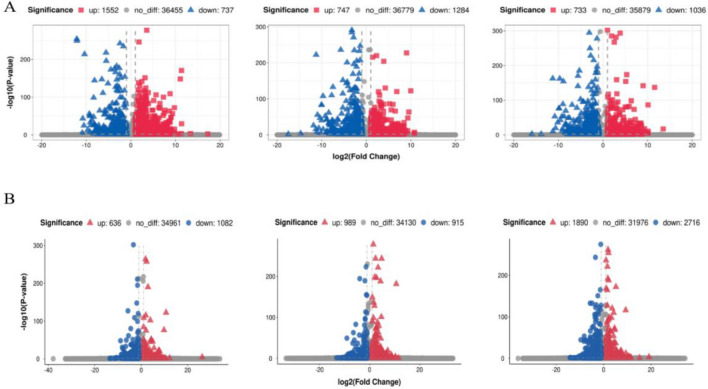
Volcano diagram of genes expression of grape and peach in three periods. (**A**) Grape ‘Pinot Noir’ and ‘Changbai No. 9’. (**B**) ‘Okubo’ and ‘Lianyungang Maotao’ From left to right are the Volcano diagrams of the gene expression of grape and peach in the young fruit stage, swelling stage, and mature stage; the abscissa is log_2_ (Fold Change), and the ordinate is -log_10_(*P*-value); up: significantly up-regulated gene, down: significantly down-regulated gene, no_diff: no significant change in gene.

**Figure 4:**
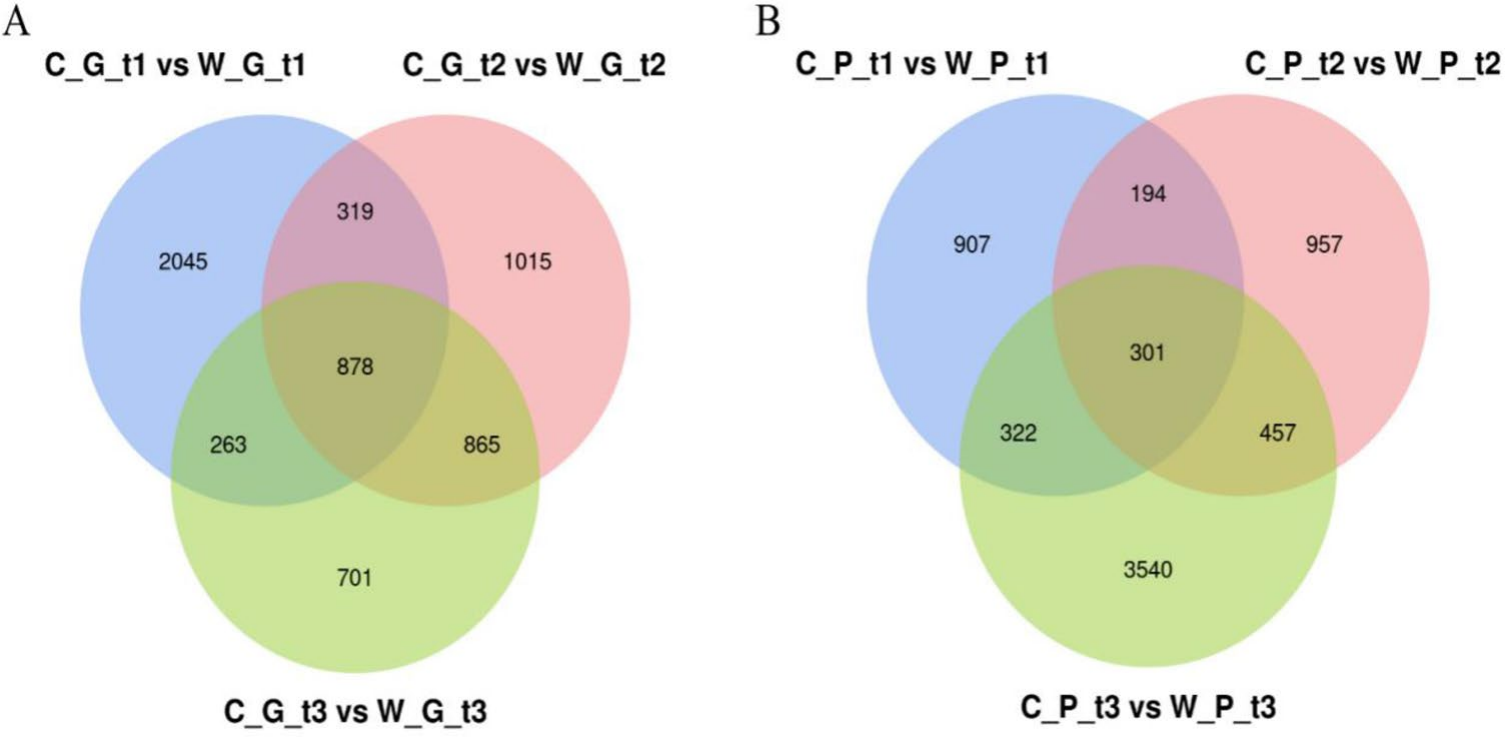
Venn diagram of differential expression of genes of ‘Pinot Noir’ and ‘Changbai No. 9’ in three periods of grapes (**A**) and peaches (**B**) respectively.

The volcano diagram of the relative changes in expression level of grapes in each period (Fig. 3A), which compares the transcripts of ‘Changbai No. 9’ to the transcripts of ‘Pinot Noir’. The results showed that in the young fruit stage, the number of up-regulated transcripts was 1,552 and the number of down-regulated transcripts was 737; in the swelling stage, the number of up-regulated transcripts was 747, and the number of down-regulated transcripts was 1284. In the mature stage, the number of up-regulated transcripts was 733, and the number of down-regulated transcripts was 1036. It can be seen that the transcripts of 'Pinot Noir' were more abundant in the young fruit stage, and more transcripts were up-regulated. In the expansion and maturity stages, number of upward adjustments is more. A total of 55,956 transcripts were expressed in ‘Pinot Noir’, and a total of 53,446 transcripts were expressed in ‘Changbai No. 9’, with 49,232 co-expressed transcripts. After the differential expression screening of the transcript expression level, it was found that the differential transcripts in the young fruit stage, the swelling stage, and the mature stage gradually decreased. There were 3505, 3077 and 2707 differential transcripts respectively (Fig. 4A), and a total of 878 common transcripts in the three periods. There were 6086 common differential transcripts and a total of 6086 non-repetitive differential transcripts.

A differential expression analysis of peach transcripts was performed (Fig. 3B). Compared with 'Lianyungang Maotao', ‘Okubo’ contained 636、989 and 1,890 up-regulated transcripts at three stages respectively, and 1082、915 and 2,716 down-regulated transcripts respectively. In the young fruit stage and the mature stage, the transcripts of ‘Okubo’ were more abundant, and there were more transcripts up-regulated. In ‘Okubo’, a total of 46,172 transcripts were expressed, and in ‘Lianyungang Maotao’, a total of 46,372 transcripts were expressed. According to the selection criteria of *P*-value < 0.05 and |Fold Change|> 1, significantly differentially expressed transcripts were screened (Fig. 4B). The results showed that the number of differentially expressed transcripts in the young fruit stage and the swelling stage were similar, 907 and 957 respectively. The number of differentially expressed transcripts in the mature stage was larger, which was 3540. There were 301 common differentially expressed transcripts in the young fruit stage, swelling stage and mature stage.

### STEM analysis of differentially expressed genes

**Figure 5:**
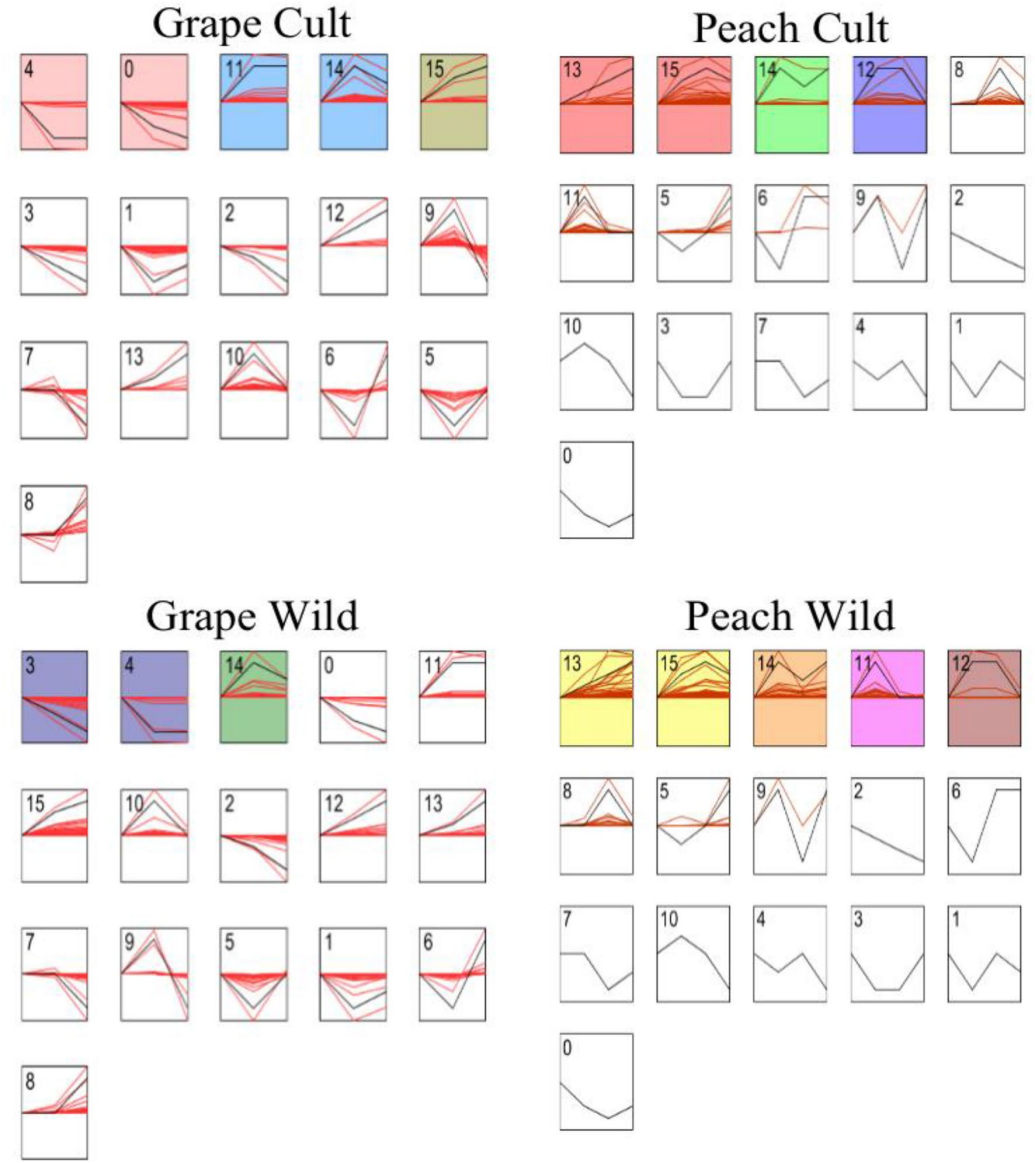
Short time-series expression miner analysis of gene expression in three periods of cultivar and wild grape and peach. Expression trends analysis of 878 differential expression genes in cultivar and wild grape species and 301 differentially expressed genes in cultivar and wild peach species. Each box represents a transcript expression pattern. The colored box indicates that there is a significant distribution of transcripts, the number in the upper left corner is the model number; the black line represents the total expression trend of the box, and the red line represents the expression trend of each gene in the box.

Time series analysis of 878 and 301 co-differentially expressed genes in grape and peach was performed by STEM software. As shown in Fig. 5, the differentially expressed genes of 'Pinot Noir' are divided into 16 modules, of which there are five modules with significant differential expression, namely modules 0, 4, 11, 14, and 15, of which module 4 contains the most genes, reached 259, and module 15 is the least, with only 42. There are 3 significant modules in ‘Changbai No. 9’, namely module 3, 4 and 14, of which module4 has the most genes (113). Module3 has 34 genes. The expression trends of the two grape species in the module are same, and the difference is mainly reflected in the expression level of the genes. In the two different significant expression modules 0, 3, 11, and 15, the expression level of 'Pinot Noir' is higher than that of ‘Changbai No. 9’. In the same significant expression module14, it was first up-regulated and then down-regulated, and the expression level of ‘Changbai No. 9’ in this module was higher.

It can be seen from Fig. 5 that the genes of ‘Okubo’ are significantly clustered in four modules (12, 13, 14, 15), and the genes of ‘Lianyungang Maotao’ are significantly clustered in five modules (11, 12, 13, 14, 15). Module14 and Module15 in ‘Okubo’ are the most representative modules. The expected distribution of genes are 14.6 and 13.3, respectively, and the actual distribution of genes are 49.5 and 45. The most representative modules in ‘Lianyungang Maotao’ are module11 and module14, the expected distribution of genes are 19.8 and 15.4, respectively, and the actual distribution are 33 and 56.5. Similar expression patterns are maintained in the common modules (12, 13, 14, 15) of ‘Okubo’ and ‘Lianyungang Maotao’. The difference between the two modules is mainly reflected in the amount of expression. In module12, the average expression level of ‘Lianyungang Maotao’ is higher, reaching 167.4, and that of ‘Okubo’ is 113.9. With module13, the expression of ‘Okubo’ was more active, reaching 52.5, and the expression of ‘Lianyungang Maotao’ was 33.8. With module14, the expression levels of ‘Okubo’ and ‘Lianyungang Maotao’ were significantly different, which was 68.9 and 37.9, respectively. With module 15, the expression levels of ‘Okubo’ were lower than that of the ‘Lianyungang Maotao’, which was 26.6 and 42.8 respectively. The significant expression module, No. 11 of ‘Lianyungang Maotao’ is not significant in ‘Okubo’. Therefore, after analyzing the function of the transcript in this module, it is found that its function is mainly concentrated in the overall composition of the membrane, chloroplast, and chloroplast thylakoid membrane, iron ion binding, chlorophyll binding, flavonoid biosynthesis process, anti-stress response and photosynthesis, etc.

### Analysis of co-expression network of differential expressed genes

**Figure 6:**
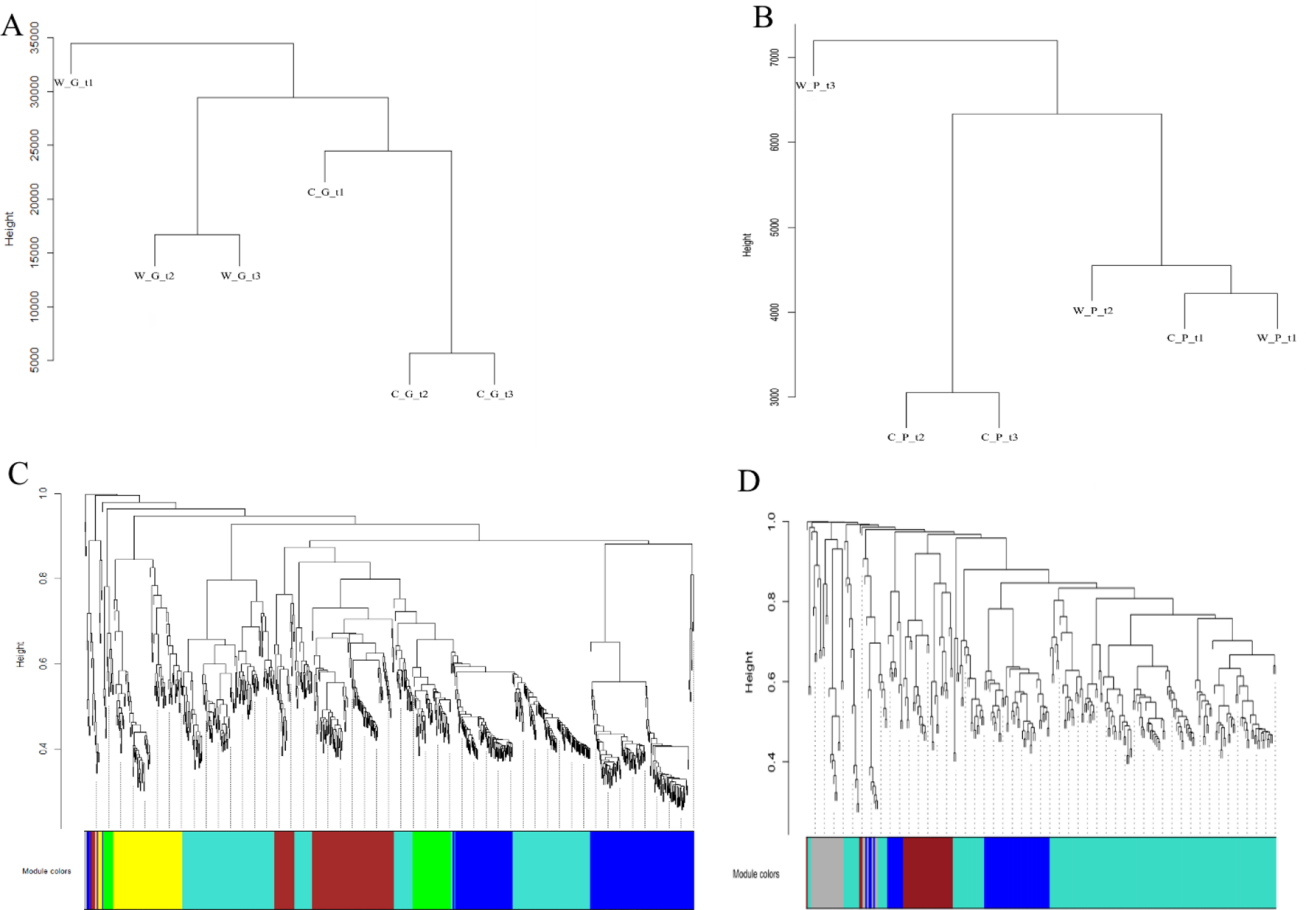
Analysis of weighted gene co-expression network of common differentially expressed genes of grape and peach. (**A**) Gene clustering dendrogram for the three periods of ‘Pinot Noir’ and ‘Changbai No. 9’. (**B**) Gene clustering dendrogram for the three periods of ‘Okubo’ and ‘Lianyungang Maotao’. (**C**) Module construction for the three periods of ‘Pinot Noir’ and ‘Changbai No. 9’. (**D**) TModule construction for the three periods of ‘Okubo’ and ‘Lianyungang Maotao’.

The 878 and 301 common differentially expressed genes of grapes and peaches were analyzed by weighted gene co-expression network analysis (WGCNA), and their subsequent co-expression modules were studied to reveal their differences. According to the expression level of the transcript, a cluster dendrogram was made for the grapes in the three periods. The expression level of ‘Changbai No. 9’ in the young fruit stage is quite different from that of other samples (Fig. 6A), while the samples in the expansion stage and mature period appeared clustered. Six modules are finally obtained with the 878 genes of grapes, of which module Turquoise, Blue, Brown, Yellow and Green contains 302, 241, 156, 104 and 70 genes respectively (Fig. 6C). The Grey module includes genes not belonging to any modules and with no function.

The function of the genes in the Turquoise module is mainly related to the overall composition of the membrane and the response to low temperature stress. The genes in the Blue module are related to the nucleus and plasma membrane. The genes in the Brown module are related to the heat stress response and cytoplasm. The genes in the Yellow module are related to the binding of metal ions and the transcript DNA template, and the Green module is related to the overall composition of the nucleus and membrane.

Analysis of the 301 common differentially expressed genes of ‘Okubo’ and ‘Lianyungang Maotao’ found that the expression level of ‘Lianyungang Maotao’ at the maturity stage (W_P_t3) was significantly different from other samples (Fig. 6B). As shown in Fig. 6D, four modules are finally formed, of which the Turquoise module contains 183 genes, the Blue module contains 56 genes, the Brown module contains 35 genes, and the Grey module with no function. After annotating the genes function in each module, it is found that the genes in the Turquoise module are mainly enriched in the cytoplasm, ATP binding and stress resistance; the genes in the Blue module are mainly concentrated in the nucleus, DNA binding and stress resistance; the genes in the Brown module are mainly enriched in the overall composition of the membrane, chlorophyll binding and photosynthesis.

### Differentially expressed genes GO function enrichment analysis

878 and 301 co-expressed genes of grape and peach were analyzed by GO enrichment. The 878 common differentially expressed genes of ‘Pinot Noir’ and ‘Changbai No. 9’ have six annotations at the Cellular component, 12 annotations at the Molecule function, and 16 annotations at the Biological process (Table 4). In terms of cell components, annotations are mainly focused on integral component of membrane, nucleus, plasma membrane, mitochondrion, golgi membrane and nucleoplasm. In terms of molecular functions, annotations mainly focus on ATP binding, zinc ion binding, DNA binding, sequence-specific DNA binding transcription factor activity, RNA binding, and protein serine/threonine kinase activity. In the biological process, annotations mainly focus on transcription, DNA-templated, regulation of transcription, defense response, protein transport, embryo development ending in seed dormancy and mRNA processing.

**Table 4:** GO function enrichment analysis of common differentially expressed genes of cultivated and wild grape species.

GO	GO term	Number	*P*-value
Cellular component	Integral component of membrane	119	2.19E − 04
	Nucleus	96	3.44E − 09
	Plasma membrane	79	2.32E − 02
	Mitochondrion	27	5.91E − 03
	Golgi membrane	9	2.77E − 02
	Nucleoplasm	2	1.59E − 02
Molecular function	ATP binding	58	3.11E − 26
	Zinc ion binding	36	1.63E − 02
	DNA binding	30	1.14E − 08
	Sequence-specific DNA binding transcription factor activity	20	2.09E − 06
	RNA binding	19	8.57E − 03
	Protein serine/threonine kinase activity	11	9.39E − 15
	Ligase activity	9	1.83E − 02
	Nucleic acid binding	3	4.76E − 03
	Phosphoprotein phosphatase activity	2	4.90E − 02
	Calmodulin binding	2	4.38E − 03
	Serine-type endopeptidase activity	1	2.13E − 02
	ADP binding	1	3.36E − 23
Biological process	Transcription, DNA-templated	38	2.00E − 08
	Regulation of transcription, DNA-templated	25	1.41E − 06
	Defense response	17	2.54E − 10
	Protein transport	8	4.18E − 02
	Embryo development ending in seed dormancy	6	2.04E − 02
	mRNA processing	4	2.47E − 03
	Plant-type hypersensitive response	3	2.26E − 04
	Metabolic process	2	3.80E − 02
	Flower development	2	4.05E − 03
	DNA replication	1	1.08E − 02
	DNA repair	1	8.21E − 05
	DNA recombination	1	2.13E − 02
	Intracellular protein transport	1	1.96E − 02
	Mitotic nuclear division	1	1.81E − 02
	Chromatin modification	1	1.87E − 02

The GO function enrichment analysis of 301 common differentially expressed genes of peaches found that there are 10 annotations at the Cellular component, 8 annotations at the Molecule function, and 4 annotations at the Biological process (Table 5). In terms of cell components, annotations are focused on integral component of membrane, nucleus,、chloroplast, plasma membrane, cytosol, mitochondrion, endoplasmic reticulum membrane, golgi apparatus, nucleolus and chloroplast stroma. In terms of molecular functions, annotations are concentrated on ATP binding, metal ion binding, DNA binding, protein serine/threonine kinase activity, zinc ion binding, RNA binding, sequence-specific DNA binding transcription factor activity and GTP binding. In terms of biological processes, annotations focus on regulation of transcription, DNA-templated, transcription, mRNA processing and protein ubiquitination.

**Table 5:** GO function enrichment analysis of 301 common differentially expressed genes of cultivated and wild peach species.

GO	GO term	Number	*P*-value
Cellular component	Integral component of membrane	28	7.89E − 06
	Nucleus	21	6.28E − 09
	Chloroplast	14	4.63E − 02
	Plasma membrane	10	7.02E − 07
	Cytosol	7	1.14E − 03
	Mitochondrion	5	1.09E − 03
	Endoplasmic reticulum membrane	3	4.55E − 02
	Golgi apparatus	3	1.80E − 02
	Nucleolus	2	2.94E − 02
	Chloroplast stroma	1	1.00E − 02
Molecular function	ATP binding	26	1.64E − 05
	Metal ion binding	11	1.05E − 03
	DNA binding	9	6.90E − 05
	Protein serine/threonine kinase activity	6	4.32E − 04
	Zinc ion binding	5	1.86E − 04
	RNA binding	4	4.88E − 03
	Sequence-specific DNA binding transcription factor activity	2	6.51E − 06
	GTP binding	1	4.67E − 02
Biological process	Regulation of transcription, DNA-templated	8	2.99E − 03
	Transcription, DNA-templated	7	7.85E − 07
	mRNA processing	1	3.08E − 02
	Protein ubiquitination	1	4.45E − 03

The differentially expressed genes of cultivated and wild materials in our study have similar items in GO functional enrichment analysis. Cell component included integral component of membrane, nucleus, plasma membrane, and mitochondrion; molecular functions included ATP binding and zinc ion binding, DNA binding, sequence-specific DNA binding transcription factor activity, RNA binding, and protein serine/threonine kinase activity; biological processes included regulation of transcription, DNA-templated, transcription, and mRNA processing.

### Differentially expressed genes KEGG pathway enrichment analysis

KEGG pathway enrichment analysis was performed on 878 and 301 common differentially expressed genes of cultivated and wild materials to explore the function of genes in the plant growth process from multiple angles. The 878 common differentially expressed genes of ‘Pinot Noir’ and ‘Changbai No. 9’ are concentrated in 103 pathways. The functions are mainly concentrated in metabolic pathways, biosynthesis of secondary metabolites, protein processing in the endoplasmic reticulum, carbon metabolism, glutathione metabolism, amino acid biosynthesis, phenylpropane biosynthesis, peroxisomes, glycolysis/glycolysis raw and pyruvate metabolism etc. According to the results of KEGG and GO analysis, a total of ten genes related to grape resistance, fruit color, peel color and fruit flavor were screened. Among them, there are five genes related to grape fruit resistance: superoxide dismutase (SOD2), glutathione S-transferase (GST), serine/threonine-protein kinase (PBS1), basic endochitinase B (CHIB) and calreticulin (CALR); fruit size is related to two genes: auxin-responsive protein (*IAA*) and ABA responsive element binding factor (*ABF*); two fruit flavor-related genes: malate dehydrogenase (*MDH1*) and pyruvate kinase (*PK*); one peel color-related gene: chalcone synthase (*CHS*).

The 301 common genes of ‘Okubo’ and ‘Lianyungang Maotao’ clustered in 51 pathways. The functions of the differentially expressed genes are mainly concentrated in metabolic pathways, flavonoid biosynthesis, tyrosine metabolism, pyrimidine metabolism, alpha-linolenic acid metabolism, photosynthesis, pyruvate metabolism, photosynthesis-antenna proteins, pantothenate and CoA biosynthesis. These pathways may be closely related to the differences in the traits of ‘Okubo’ and ‘Lianyungang Maotao’ in peel color, fruit weight, sugar and acid content. Combining the results of KEGG pathway enrichment analysis and GO function annotation results, it was found that 12 genes were related to peach fruit resistance, peel color and fruit growth. Among them, eight genes are related to fruit resistance: polyphenol oxidase, peroxidase, glutathione S-transferase (*GST*), chitinase, 12-oxophytodienoic acid reductase (*OPR*), light-harvesting complex II chlorophyll a/b binding protein 4 (*LHCB4*), 9-cis-epoxycarotenoid dioxygenase (*NCED*) and alcohol dehydrogenase class-P (*ADH1*); there are three genes related to fruit color: chalcone synthase (*CHS*), phenylalanine ammonia-lyase (*PAL*) and bifunctional dihydroflavonol 4-reductase/flavanone 4-reductase (*DFR*), and one gene related to fruit growth: SAUR family protein (*SAUR*). Considering KEGG annotations in grapes and peaches: metabolic pathways and pyruvate metabolism are the common pathways, which may explain the differences between cultivated and wild fruit trees.

## Discussion

‘Pinot Noir’ is a world-renowned grape wine variety, and ‘Changbai No. 9’ is widely planted in production as a wild grape strain with strong cold resistance. The cultivated and wild species of grapes and peaches have similar differences in fruit horizontal and vertical diameter and single fruit weight: the horizontal diameter, vertical diameter and single fruit weight of the cultivated species are greater than those of the wild species. The horizontal diameter of ‘Pinot Noir’ in the mature stage is 1.14 times that of ‘Changbai No. 9’, and the longitudinal diameter is 1.24 times. The difference in single fruit weight is the most significant, reaching 1.51 times. The horizontal diameter of ‘Okubo’ in the mature period is 1.78 times that of ‘Lianyungang Maotao’; the longitudinal diameter is 1.65 times, and the difference in single fruit weight is 5.15 times.

In this study, an auxin-responsive gene related to the difference in agronomic traits of cultivated and wild fruits was identified in peaches: AUR family protein (*SAUR*). Auxin plays a key regulatory role in plant cell division, elongation and plant growth and development^13–15^. The *SAUR* gene family is a plant-specific gene family and it can interact with calmodulin to affect the relationship between the calmodulin secondary signaling system and the auxin signaling pathway^16^. Previous studies have shown that during plant growth and development, auxin-responsive genes have a close regulatory effect on fruit development by regulating the balance of auxin in the plant body. In this study, two genes related to fruit size were found: auxin-responsive protein (*IAA*) and ABA responsive element binding factor (*ABF*). *AUX_IAA* and *ARF* can recognize the auxin response element (Aux REs), thereby regulating auxin-responsive genes^17^. The GO annotation results of the differential transcripts of ‘Pinot Noir’ and ‘Changbai No. 9’ contain ATP binding, zinc ion binding, protein transport and other functions, which can promote the transportation and absorption of nutrient elements in plants, thereby affecting plant growth and fruit swelling and development.

Fruit flavor is affected by plant genetic factors, environmental factors, and cultivation techniques^18^. ‘Pinot Noir’ and ‘Okubo’ are grown in artificial greenhouses, while ‘Changbai No. 9’ and ‘Lianyungang peach’ are grown in nature, therefore cultivated and wild grapes and peaches have big differences in fruit flavors, which are reflected in the content of soluble sugars and organic acids. In this study, the sugar content of cultivated varieties was higher than that of wild materials (Fig. 1); this was attributed to the differences caused by different growth conditions between cultivated and wild varieties. These differences were also reflected at the gene level, which also proved that in the process of cultivation, domestication and utilization of grapes, the direction of increasing the ratio of sugar and acid has been developed.

The flavor quality of peach fruit is affected by the soluble sugar, organic acid, sugar-acid ratio and volatile aromatic substances in the fruit. The sweetness of the fruit is most affected by fructose, followed by sucrose, glucose and sorbitol^19^. In mature peach fruit, sucrose is the main component of its sugar component, accounting for 40–85% of the total sugar content^20^, which is consistent with the results of this study, ‘Okubo’ and ‘Lianyungang Maotao’ sucrose content accounted for 58.4% and 62.6% of the total soluble sugars, respectively. There was a big difference in sugar acid content and sugar acid ratio between ‘Okubo’ and ‘Lianyungang Maotao’. Through the identification of 878 common differentially expressed transcripts of 'Pinot Noir' and ‘Changbai No. 9’ in three periods, it was found that two genes were related to fruit flavor: malate dehydrogenase (*MDH1*) and pyruvate kinase (*PK*), and participated in glycolysis/gluconeogenesis and MAPK signaling pathways. Studies have shown that pyruvate kinase can determine the final product of glycolysis, so as to cultivate plants that are more in line with needs^21^.

The color of grape peel has an important impact on its economic value. At present, many studies have shown that the content and type of anthocyanins play a decisive role in the color of grape peel. Anthocyanins are glycoside derivatives of anthocyanins. The content of anthocyanins in grape fruits is higher and contains many kinds of anthocyanins^22^. Anthocyanidins exist in the vacuoles of plant cells and are synthesized in the cytoplasm from flavonoids through the shikimate pathway^23,24^. Chalcone synthase is a key enzyme in the synthesis of flavonoids, and its expression has been shown to be closely related to the accumulation of anthocyanins^25^. After studying the color changes of the fruits of ‘Pinot Noir’ and ‘Changbai No. 9’, it was found that chalcone synthase (*CHS*), a gene related to anthocyanins, was enriched in pathways such as flavonoid biosynthesis and monoterpene biosynthesis. The fruit colors of ‘Pinot Noir’ and ‘Changbai No. 9’ in the young fruit stage are both green, and there is a big difference in the swelling stage. The fruit of ‘Pinot Noir’ remains green, while the color of ‘Changbai No. 9’ is purple and black.

In terms of peel color, there is no obvious difference between the young fruit stage and the swelling stage of the ‘Lianyungang Maotao’ fruit peel, but the peel color of the 'Okubo' is significantly redder at the mature stage. There are 301 common differentially expressed genes between ‘Okubo’ and ‘Lianyungang Maotao’. After analysis, three genes related to peel color were found: chalcone synthase (*CHS*), phenylalanine ammonia-lyase (*PAL*) and bifunctional dihydroflavonol 4-reductase/flavanone 4-reductase (*DFR*). Research shows that *CHS* in peaches found that inhibiting the expression of *CHS* gene caused the anthocyanin metabolic pathway to shift to the direction of chlorogenic acid and complexes, proving that *CHS* gene is closely related to the metabolism of flavonoids^26^. The expression levels of *CHS* gene-related transcripts of ‘Okubo’ and ‘Lianyungang Maotao’ also showed a significant difference of 5 times, and the expression levels at the mature stage were 349 and 69.3. Research shows that the anthocyanin biosynthesis of peaches of different colors and showed that *PAL* gene may affect the anthocyanin metabolism pathway in peach fruits^27^. The *DFR* gene plays a key role in the formation of anthocyanins^28^. *DFR* can use three different substrates (*DHK*, *DHQ* and *DHM*) to synthesize different colors^29^.

Grapes are susceptible to infection by pathogens such as anthracnose, downy mildew, white rot and black pox. They also face abiotic stresses such as drought and ultraviolet radiation, which greatly affect the yield of grapes. Studies by Carvolho^30^ and Daldoul^31^ have shown that wild grapes have important research significance in biological/abiotic resistance. Salicylic acid accumulates in a stress environment, and its signaling pathway is related to plant resistance^32^. Under abiotic stress, salicylic acid still has an effect on resistance^33,34^. In the process of plants resisting stress, antioxidant systems play a key role, such as: superoxide dismutase (*SOD*), peroxidase (*POD*), catalase (*CAT*)^35^. The KEGG enrichment pathway analysis of 878 common differential genes found that the metabolic pathways related to biotic/abiotic stress are mainly concentrated in: peroxisomes, glutathione metabolism, phagosomes, interactions between plants and pathogens and other pathways. Five genes related to resistance of grape berries were screened: superoxide dismutase (*SOD*), glutathione S-transferase (*GST*), serine/threonine-protein kinase (*PBS1*), basic endochitinase B (*CHIB*) and calreticulin (*CALR*). SOD has the ability to eliminate superoxide anion free radicals and participates in response pathways under various stress environments of plants. Studies have shown that glutathione S-transferase (*GST*), glutathione peroxidase (*GPX*), and glutathione reductase (*GR*) are the key enzymes that glutathione participates in plant defense mechanisms^36^. Known studies have shown that *PBS1* participates in Pep-induced plant defense response signal transmission. After analyzing the expression of resistance-related transcripts, it was found that the average TPM value of the resistance-related transcripts of ‘Changbai No. 9’ was 798.9, and the average TPM value of the resistance-related transcripts of ‘Pinot Noir’ was 156.9. This result is consistent with previous studies and proves that the anti-stress ability of ‘Pinot Noir’ still has a large scope for domestication and improvement, and there are resistance genes worth studying in ‘Changbai No. 9’.

Peach has become the most widely used rootstock species in China due to its excellent resistance^37^. This study screened the common differential genes of ‘Okubo’ and ‘Lianyungang Maotao’ and found eight fruit resistance-related genes: polyphenol oxidase, peroxidase, glutathione S-transferase (*GST*), chitinase, 12-oxophytodienoic acid reductase (*OPR*), light-harvesting complex II chlorophyll a/b binding protein 4 (*LHCB4*), 9-cis-epoxycarotenoid dioxygenase (*NCED*) and alcohol dehydrogenase class-P (*ADH1*). The GO function annotation contains multiple resistance-related annotations: response to wounding (GO:0009611) response to nematode (GO:0009624) and response to insect (GO:0009625). The polyphenol oxidase gene has been shown to have an effect on plant resistance^38^. The chitinase enzyme^39,40^, affects and degrades chitin in the fungal cell wall, and acts as an elicitor to regulate the defense response of the plant. After analyzing the expression levels of resistance genes in ‘Okubo’ and ‘Lianyungang Maotao’, it was found that their expression levels were quite different: the average expression level of chitinase gene-related transcripts in ‘Okubo’ in the three periods was 30.53, while the expression level of the more resistant ‘Lianyungang Maotao’ was 102.93, indicating that ‘Okubo’ still has a great potential for improvement and needs further cultivation and domestication.

It has been proved that chalcone synthase (*CHS*), glutamate S-transfer (*GST*) and malate dehydrogenase (*MDH1*) genes play an important role in the growth of grapes and peaches. The gene expression pattern of infected grapes was detected by qRT-PCR. It was found that the highly expressed *CHS* gene could enhance the ability of grape leaves to resist gray mold and downy mildew, which was consistent with the results of this study (the average expression amount of related genes in cultivated grapes was 16.35, while the average expression amount of related genes in wild grapes was 63.07)^41^. In peaches, the *CHS* gene regulates the metabolism of flavonoids, affects the synthesis of anthocyanin glycosides, quercetin glycosides, and chlorodermin, and has an important impact on peel color^42^. The correlation between *GST* gene function deletion allele and white peel has been verified by genetic markers in peach^43^. The correlation between *MDH1* gene in grape and malic acid content in fruit has been proved by RNA seq and qRT PCR^44^.

## Methods

### Experimental material collection

The cultivated plants ‘Lianyungang Maotao’ and ‘Okubo’ were healthy plants cultivated for 5 years by Jiangsu Academy of Agricultural Sciences (Nanjing Jiangsu Province, China) and ‘Changbai No. 9.’ and ‘Pinot Noir’ for 8 years by Zhengzhou Fruit Tree Institute (Zhengzhou City, Henan Province, China) respectively.

Grape fruit materials are collected in the young fruit period (June 6), swelling period (July 17) and ripening period (July 30). Peach fruit materials are collected in the young fruit period (May 6), the swelling period (June 24) and maturity period (cultivated peach: July 17; wild peach: August 15) (Table 1). Selected normal fruits and leaves from branches with similar height from the ground and free of disease and insect marks. At least 10 fruits of each variety were mixed in each period as a sample. The sample was frozen in liquid nitrogen immediately after collection, and then stored in ultra-low temperature refrigerator at minus 80 degrees Celsius.

The plant materials involved in this experiment had been approved by the unit before collection, and the research was in compliance with local policies and regulations.

### Determination of fruit transverse and longitudinal diameter and single fruit weight

The horizontal diameter, vertical diameter and single fruit weight of grapes were measured by vernier calipers and electronic balance. And average of the horizontal diameter, vertical diameter and single fruit weight of the fruit at different growth stages were made according to the measured data.

### Measurement of fruit soluble sugar and organic acid

Took two grams of fruits stored in a liquid nitrogen environment and fully grind them in the mortar, then add the extract, heat treatment, ultrasonic extraction and centrifugation in a 37 °C water bath, and collect the supernatant in a volumetric flask, performed three repetitions. After constant volume, used a rotary evaporator to process and add 1 mL of ultrapure water. The extracted liquid was filtered through a chromatographic column SEP-C18 column (Waters, WAT021515) and Sep-Pak filter.

The soluble sugar indicators included sorbitol, sucrose, fructose and glucose. The soluble sugar content in the fruit was determined by high performance liquid chromatography. The equipment and parameters were set as followed: Carbohydrate column (Transgenomic COREGET-87C) and guard column (Transgenomic CARB Sep Coregel 87C cartridge);column setting temperature: 85 °C, reference cell temperature: 35 °C; flow rate setting: 0.8 mL/min; ultrapure water; injection volume 5 μm. Organic acid indicators include quinic acid、citric acid、malic acid、oxalic acid and shikimic acid. The equipment and parameter settings are as follows: Zobar SB-Aq column; flow rate setting: 0.7 mL/min; 20 mmol/l disodium hydrogen phosphate buffer; 2% methanol 98%; column temperature setting: 35 °C; water UV detector: wavelength 210 nm; injection volume 5 μm. Calculate the soluble sugar and organic acid content based on the output data.

### RNA extraction and cDNA library preparation

RNA samples were extracted by the cetyltrimethyl ammonium bromide method (CTAB)^45^. The pulp tissue samples of grapes and peaches were added to CTAB solution and liquid nitrogen for grinding, and approximately 1 mg was extracted respectively. According to the Illumina^®^TruSeq™RNA sample preparation process, the extracted RNA sample was used to prepare a cDNA library, first strand cDNA was synthesized using random hexamer primer and second strand cDNA synthesis was subsequently performed using DNA Polymerase I and RNase H.

### Transcriptome sequencing, assembly, annotation and quantification process

Library quality was assessed on the Agilent Bioanalyzer 2100 system and then sequenced with Illumina platform and paired-end reads were generated. The adaptor sequences and low-quality sequence reads were removed from the raw sequences. Download grape and peach genome data and annotations from NCBI (National center for biotechnology information) database. These clean reads were then mapped to reference genome by Tophat tool^46^. TransDecoder was used first to identify potential coding regions (CDS) in the sequences, Diamond software was then used for sequence similarity comparison. After the comparison was completed, Hmmer was used to search for protein domains, and finally the above annotation informations were integrated through the Trinotate annotation tool. In this study, Kallisto^47^ data quantification software was used to calculate gene expression by TPM (Transcripts per kilobase million) standardized method (https://pachterlab.github.io/kallisto/).

### Analysis and identification of differentially expressed genes

The identification of differentially expressed genes were identified by the R program platform DEGseq package^48^. DEGseq was used to analyze the fruit transcript expression levels of samples. The threshold of significantly differentially expressed transcripts and false positive rates of grape and peach were | Fold Change |> 1 and *P*-value < 0.01, | Fold Change |> 2 and *P*-value < 0.05, respectively.

Short time-series expression miner (STEM) (http://www.cs.cmu.edu/~jernst/stem/) was a JAVA-based gene expression trend analysis program, which could be logarithmic perform cluster analysis and visualization of gene expression data at each time point, and used to perform Gene ontology (GO) annotations on genes with the same expression pattern^49^. The differential transcript expression levels of wild grapes ‘Changbai No. 9’, cultivated grapes ‘Pinot Noir’, cultivated peaches ‘Okubo’ and wild peaches ‘Lianyungang Maotao’ at the same stage were input into expression profile data analysis. The software analyzes the expression trends of the three periods.

### Functional analysis of differentially expressed genes

Weighted gene correlation network analysis (WGCNA) can organize genes with the same expression pattern into modules, and association analysis between the modules and phenotypic data were performed to mine potential key genes^50^. In this study, 878 and 301 differential genes between wild grape ‘Changbai No. 9’ and cultivated grape ‘Pinot Noir’, cultivated peach ‘Okubo’ and wild peach ‘Lianyungang Maotao’ at three periods as input data respectively. The soft threshold power was screened and the genes were divided into modules.

Gene ontology^51^ annotation analysis includes three parts: Cellular component, Molecular function and Biological process. Gene ontology describes the characteristics of genes and gene products through these three aspects, and GO annotation analysis was widely used in gene function annotation as a unified tool in biology. The Kyoto Encyclopedia of Genes and Genomes (KEGG) can assign functional meanings to genes and genomes at the molecular level and understand the functions of biological systems^52^. This study used GO and KEGG to annotate the differential expression transcripts of cultivated grape ‘Pinot Noir’ and wild grape ‘Changbai No.9’, cultivated peach ‘Okubo’ and wild peach ‘Lianyungang Maotao’ to further understand the function and pathway differences between cultivated and wild fruit trees.

## Data Availability

Raw reads of the experiment are submitted to NCBI SRA database, the accession number of the project is PRJNA792624, https://www.ncbi.nlm.nih.gov/bioproject/PRJNA792624.
